# Prevalence, Characteristics, and Epidemiology of Microbial Hand Contamination Among Minnesota State Fair Attendees (2014)

**DOI:** 10.3389/fpubh.2020.574444

**Published:** 2020-12-16

**Authors:** Meghan R. Mason, Bozena M. Morawski, Ruby L. Bayliss, Fatuma M. Noor, Sagal H. Jama, Connie L. Clabots, James R. Johnson

**Affiliations:** ^1^Public Health Department, Henrietta Schmoll School of Health, Saint Catherine University, Saint Paul, MN, United States; ^2^Division of Epidemiology and Community Health, School of Public Health, University of Minnesota, Minneapolis, MN, United States; ^3^Minneapolis VA Health Care System, Minneapolis, MN, United States; ^4^Division of Infectious Disease and International Medicine, School of Medicine, University of Minnesota, Minneapolis, MN, United States

**Keywords:** microbial diversity, prevalence, hand hygiene, epidemiology–descriptive, community gatherings

## Abstract

**Background:** Many antimicrobial-resistant infections are community-acquired, yet community carriage of microorganisms by healthy individuals is poorly characterized. We assessed microorganism carriage on the hands of Minnesota State Fair attendees and explored associated factors.

**Methods:** Minnesota State Fair attendees (in 2014) from households with ≥2 members (≥1 member being <19 years old [a child]) were eligible to participate. Participants provided biological samples via a hand plating technique and completed a questionnaire on factors potentially related to microorganism carriage. Using presumptive taxonomic identifications and disk-diffusion-determined resistance phenotypes, hand-culture isolates were classified by microbial type; types were grouped into four broad categories based on inferred pathogenicity and consistency with the skin microbiota. Descriptive statistics, *X*^2^ tests, and generalized linear mixed-effects models were used to explore associations between survey and culture data.

**Results:** We enrolled 206 participants from 82 households during 2 days; 50% of subjects were children. Overall, 99.5% (205/206) of hand samples yielded microorganisms. Most were non-pathogenic, whether skin microbiota (98.5% of participants) or non-skin microbiota (93.2% of participants). Only 2.4% (5/206) of samples yielded antibiotic-resistant bacteria. Children were more likely than adults to carry potentially pathogenic (OR = 3.63, 95% CI: 1.66–7.93) and presumably non-pathogenic (OR = 6.61, 95% CI: 1.67–26.15) non-skin microorganisms.

**Conclusions:** Large community gatherings can serve as efficient sites for estimating the prevalence of microorganism carriage. A small proportion of participants carried antimicrobial-resistant pathogens on their hands; most carried non-pathogenic microorganisms, and no exposures specific to the state fair were associated with microorganism carriage.

## Introduction

In 1846, Ignaz Semmelweis hypothesized that infectious particles could be transferred from person to person via a clinician's hands (1). Hands have subsequently been implicated in innumerable infections, with transmission occurring both directly (person-to-person) and via contaminated surfaces (2–5). Today, hospital infection preventionists prioritize proper hand hygiene performance by healthcare workers (6, 7). Studies of pathogen spread within hospitals have documented every location a clinician's hand touches in a day and assessed whether “fist bump” greetings would be less transmission-prone than traditional handshakes (5, 8). Yet, each year in the United States an estimated 1.7 million hospital-acquired infections occur, resulting in 99,000 deaths—many of which are attributable to inadequate hand hygiene (9). These infections account for over $9.8 billion in healthcare expenses (10).

Most infections, however, are community-acquired (11–14). Several studies that sampled the built environment have detected pathogens—especially antimicrobial-resistant bacteria—in community locations, including computer labs, public transit systems, and on gym equipment (15–18). Although this identifies possible indirect transmission routes for community-acquired infections it does not address human carriage. Identifying which microorganisms are carried by individuals in the community is important to understand the circulation and potential transmission of pathogens in a community setting.

The annual Minnesota State Fair, which hosts approximately 2 million attendees of all ages over 12 days each August (19). Despite the Fair's location in the Minneapolis-St. Paul metropolitan area, it attracts urban and rural Minnesotans alike (20). In this study we attempted to assess the distribution of microorganisms, including potential pathogens and antimicrobial-resistant organisms, on the hands of State Fair attendees, who represent a subset of Minnesota residents. We also estimated associations between specific human behaviors and microorganisms, to identify possible ways to reduce pathogen carriage. Among the behaviors assessed were the frequency and methods of handwashing, known to reduce infection with many forms of pathogens (21, 22). Additionally, we asked participants about sites they visited at the fair, particularly animal buildings and whether they touched the animals, as it is well-established that contact with animals contribute to pathogen transmission (23). We also sought to understand potential exposures from participants' behaviors outside of the Fair that could expose them to pathogens including gardening (24), gyms and team sports (25–27), public transportation (28), and employment as a daycare (29) or healthcare provider (30–32) or with livestock (33).

## Methods

We enrolled participants in this cross-sectional survey at the Minnesota State Fair. The primary outcome was the presence of specific microorganisms on participants' hands, as assessed by contact plating. We collected data on participants' demographic characteristics, handwashing behaviors in and outside of the home, and activities while at the fair. The consent procedures in this study were approved by the University of Minnesota Institutional Review Board (No. 1406M51101).

### Participant Selection

Participants were enrolled on August 31 and September 1, 2014 in the University of Minnesota “Driven to Discover” research building at the Minnesota State Fair. The study was publicized prior to the fair on a University of Minnesota website, which listed the study objectives, eligibility criteria, and location and times of data collection.

At the fair, study staff greeted potential participants as they entered the research building. For individuals who indicated an interest in participating, study staff screened for eligibility and elicited oral informed consent or assent, as appropriate. Eligibility criteria included: having ≥2 family members at the fair who were willing to participate, ≥1 of whom was <19 years old; family members living together for ≥30 days prior to enrollment; and age ≥3 and ≤ 70 years. Participation was incentivized via a drawing for one of six prize packages, to be mailed to the winners after the fair's conclusion.

### Specimen Collection and Processing

For hand sampling, participants placed their dominant hand directly on a tryptic soy agar plate containing 5% sheep blood (Becton Dickinson, Franklin Lakes, New Jersey) according to a standardized technique [S1]. Study staff used a sterile plastic rod to distribute the sample across the agar plate. Plates were refrigerated overnight at 2°C and transported to the laboratory the following morning.

In the laboratory, plates were incubated overnight at 37°C. Any colonies were assessed for morphology (color, shape, texture, and hemolysis), and one colony each for up to three predominant morphotypes was selected for gram-staining. Gram-positive cocci were assessed for coagulase and catalase production. For catalase-positive, coagulase-positive organisms (presumptive *Staphylococcus aureus*), CHROMagar plates (Becton Dickinson, Franklin Lakes, New Jersey) were used to differentiate methicillin-susceptible from methicillin-resistant *S. aureus* (MSSA and MRSA). Gram-negative bacilli (excluding coccobaccilli) were examined for lactose fermentation and oxidase production. *Pseudomonas aeruginosa* were identified with colonial morphology on T7 agar followed by an oxidase positive result. Identification of Lactobacillus after gram-staining was the presence of alpha hemolysis and a catalase negative result. Species identification was attempted for other gram-negative fermenting bacilli including *Stenotrophomas* and other *Pseudonomas sp*. using the API-20E system (34–36) (bioMerieux, Inc., Hazelwood, MO), and antibiotic susceptibility was determined using a standardized disk diffusion method (37), and breakpoints from the Clinical and Laboratory Standards Institute (CLSI).

The resulting identifications were used to classify the isolates initially into 15 different microbial types based on presumptive identity and relevant resistance phenotypes (Table 1). These 15 types were further collapsed, arbitrarily, into four larger, quasi-homogenous categories for ease of presentation and quantitative analysis. Categories were based on a combination of the organism's (i) likelihood of occurring in the skin microbiota and (ii) potential pathogenicity in healthy hosts, vs. only in immunocompromised hosts. Presumed non-pathogenic skin (NPS) microorganisms included diphtheroids, coagulase-negative staphylococci, non-beta-hemolytic streptococci, *Micrococcus* spp., and yeast. Presumed non-pathogenic, non-skin (NPNS) microorganisms included putative *Bacillus* spp., putative *Lactobacillus* spp., non-hemolytic catalase-positive Gram-positive bacilli, Gram-negative coccobacilli, *Pseudomonas* spp., *Stenotrophomonas maltophilia*, other non-fermenting, oxidase-positive, Gram-negative bacilli, and molds. Potentially pathogenic skin (PPS) microorganisms included beta-hemolytic streptococci, MSSA, and MRSA. Potentially pathogenic non-skin (PPNS) microorganisms included oxidase-negative, non-fastidious Gram-negative bacilli.

**Table 1:** Microorganisms recovered from the hands of 206 Minnesota State Fair attendees (2014).

**Category**	**No. samples (% of 206)**	**Type**	**No. samples (% of 206)**
Presumed non-pathogenic skin (NPS) microorganisms	203 (98.5)	Diphtheroids	202 (98.1)
		Coagulase-negative staphylococci or *Micrococcus* spp.	88 (42.7)
		Non-beta-hemolytic streptococci	19 (9.2)
		Yeast	2 (1.0)
Presumed non-pathogenic, non-skin (NPNS) microorganisms	192 (93.2)	Putative *Bacillus* spp.	180 (87.4)
		Putative *Lactobacillus* spp.	1 (0.5)
		Non-hemolytic, catalase-positive Gram-positive bacilli	4 (1.9)
		Gram-negative coccobacilli	20 (9.7)
		*Pseudomonas* spp.	3 (1.5)
		*Stenotrophomonas maltophilia*	3 (1.5)
		Other non-fermenting, oxidase-positive, Gram-negative bacilli	3 (1.5%)
		Molds	106 (51.5)
Potentially pathogenic skin (PPS) microorganisms	2 (1.0)	Methicillin-susceptible *Staphylococcus aureus*	1 (0.5)
		Methicillin-resistant *S. aureus*	1 (0.5)
Potentially pathogenic non-skin (PPNS) microorganisms	39 (18.9)	Oxidase-negative, non-fastidious Gram-negative bacilli	–

### Questionnaire

Study staff interviewed participants using REDCap electronic data capture tools (38). The questionnaire captured (i) demographic variables: self-identified gender, race, ethnicity, age (categorized as <19 years [child] vs. ≥19 years [adult]), educational level, family household income, residence in the seven-county metro area (as determined by ZIP code), and housing type; (ii) possible exposures and activities at home or at the State Fair that might influence hand carriage of specific microorganisms; and (iii) hygiene and health-related variables [S2]. Assessed home exposures included animal ownership, recreational activities, events attended in the past week, and regular employment type and volunteer activities. Assessed State Fair exposures included sites the participant had visited since arrival at the fair, which such site immediately preceded study enrollment, and whether the participant had touched any animals while at the fair. The health and hygiene-related questions addressed hand-washing behaviors (when, where, and how hand hygiene was performed most recently), and topical skin product use (Table 2).

**Table 2:** Participant characteristics of 206 Minnesota State Fair attendees (2014).

	**Total No. (% of 206)**
**Adult/Child Status**	
Child: <18 years old Adult: ≥18 years old	103 (50.0) 103 (50.0)
**Gender**	
Female Male	134 (65.1) 72 (34.9)
**Race**	
Asian Black/African American White More than one race Unknown/not reported	5 (2.4) 3 (1.5) 191 (92.7) 5 (2.4) 2 (0.9)
**Ethnicity**	
Hispanic Non-Hispanic	5 (2.4) 201 (97.6)
**Residence (*****n*** **=** **202)**	
Seven-county Metro Area Other	196 (97.0) 6 (3.0)
**Sites visited /Activities at the fair**	
*Animal building* *Restrooms* *Food vendor* *Touched animals*	68 (33.0) 115 (55.8) 150 (72.8) 45 (21.8)
**Own a pet**	150 (72.8)
**Time since last handwashing (*****n*** **=** **203)**	
<2 h ≥2 h	131 (64.2) 72 (35.8)
**Most recent handwashing was at the fair**	116 (56.3)
**Most recent handwashing method (*****n*** **=** **203)**	
Soap and water Alcohol-based sanitizer Other	157 (77.3) 36 (17.7) 10 (5.0)
**Handwashing frequency per day (*****n*** **=** **199)**	
≤ 5 times 6–10 times >10 times	79 (39.7) 71 (35.7) 49 (24.6)
**Self-graded general handwashing**^†^	
A B C D F	72 (35.0) 96 (46.6) 28 (13.6) 7 (3.4) 3 (1.5)
**Self-graded handwashing today (at the State Fair) (*****n*****=205)**^†^	
A B C D F	59 (28.8) 79 (38.5) 43 (21.0) 15 (7.3) 9 (4.4)
**Applied a topical skin product**	99 (48.1)
**In the past week they…**	
*Participated in individual sports* *Worked out at a gym or did team sports* *Attended another social or community event* *Swam in a pool*	87 (42.2) 83 (40.3) 69 (33.5) 45 (21.8)
*Swam in a lake* *Gardened* *Used public transit* *Outdoor recreation (hike/camp)*	24 (11.7) 2 (1.0) 167 (81.1) 8 (3.9)
**Self or child in daycare**	27 (13.1)
**Have occupation/regular volunteer activity**	
(includes adults and children)	127 (61.7)
**During occupation/regular volunteer activity, they…**	
*Work with livestock* *Work in clinic* *Work in daycare or childcare facility* *Wear gloves regularly*	4 (3.1) 11 (8.7) 33 (26.0) 19 (15.0)
**Adult-specific variables (*****n*** **=** **103)**	
**Annual Household Income categories**	
< $50,000 $50,000–$74,999 $75,000–$99,999 $100,000–$149,999 $150,000+	25 (24.3) 16 (15.5) 18 (17.5) 24 (23.3) 20 (19.4)
**Education level**	
High school graduate Some college Technical/Associate degree Bachelor's degree Graduate or professional school	4 (3.9) 16 (15.5) 7 (6.8) 42 (40.8) 34 (33.0)
**Dwelling type**	
Single-family home Apartment or townhome	90 (87.4) 13 (12.6)

*Italics indicate non-mutually exclusive responses*.

† *Participants were asked, “If you had to give yourself a grade for handwashing practices in general/today, you would say…”. They then assigned themselves a letter grade on the scale A–F*.

### Statistical Analyses

The prevalence of the 15 microbial types and corresponding four microorganism categories was used to describe the overall diversity of microorganisms present. We used cross-tabular analyses of *X*^2^ and Fisher's exact tests to identify statistical associations between these taxa and questionnaire responses. Given the exploratory nature of the study, tests were not adjusted for multiple comparisons. We excluded from type-specific analyses any microbial types identified in <3 samples (*n* = 4), leaving 10 type-specific analyses (Table 1). We also evaluated associations between each of the four microorganism categories in Table 1 and conducted an additional analysis of questionnaire responses vs. antibiotic-resistant organisms (regardless of type or category).

For the four microorganism categories and the combined antibiotic-resistant organisms, we further evaluated associations with questionnaire responses using univariable generalized linear mixed-effects models, with a binomial distribution and a logit link (lme4 package in R, version 3.4.0). A household-level random intercept was included to account for correlation among family members. For microorganisms associated with at least one questionnaire variable, we further analyzed thee associations in multivariable mixed-effects models. Multivariable models were constructed for each microorganism category using the statistically significant variables from the univariable models, and then examined in the presence of the each of the remaining questionnaire variables. For variables associated with a particular microbial category, odds ratios (OR) and 95% confidence intervals (CI) were calculated from regression models using standard methods (39).

## Results

### Participant Characteristics

During the 2-day enrollment period we enrolled 206 State Fair attendees (103 adults, 103 children), from 82 households; each participant contributed a hand culture. Of the participants, 65.1% (*n* = 134) were female, 92.7% (*n* = 191) were white, and 97.1% (*n* = 201) were non-Hispanic. Most lived in the seven-county metro area (97.0%, *n* = 196) and were pet owners (72.8%, *n* = 150). Of the participants, 81.1% (*n* = 167) used public transportation within the past week, 60.3% (*n* = 120), usually washed their hands ≥6 times per day, and 48.1% (*n* = 99) had applied a topical skin product (e.g., makeup, sunscreen, insect repellent) the day of study participation. All adults (100.0%, *n* =103) and some children (23.3%, *n* = 24) were employed or had regular volunteer activities. Most adults had a bachelor's degree or higher (73.8%, *n* = 76), an annual household income >$50,000 (75.7%, *n* = 78), and owned a single-family home (87.4%, *n* = 90). Participants were less likely to assign themselves an “A” or “B” grade (good) on handwashing at the State Fair (67.3%) than with their usual practice (81.6%) (Table 2).

Participants' activities at the State Fair included, eating from food vendors (78.2%; *n* = 150), using public restrooms (55.8%; *n* = 115), visiting animal buildings (33.0%; *n* = 68), and touching animals (21.8%; *n* = 45). Among all participants, the most recent hand washing involved soap and water (77.3%; *n* = 157) and had occurred at the fair (in a restroom or at a handwashing station; 56.3%; *n* = 116). Common behaviors outside of the fair in the past week included use of public transit (81.1%, *n* = 167), participation in individual sports (42.2%, *n* = 87), gym use or team sports (40.3%, *n* = 83) and attendance at other social or community events (33.5%, *n* = 69).

### Microbiological Samples

Of the 206 hand samples, 205 (99.5%) were culture-positive. The most commonly identified microbial type was diphtheroids (98.1%, *n* = 202), followed by putative *Bacillus* spp. (87.4%, *n* = 180) and molds (51.5%, *n* = 106) (Table 1). Presumed non-pathogens, whether or not typically skin-associated (i.e., NPS and NPNS microorganisms), occurred in 98.5 and 93.2% of samples, respectively. By contrast, potential pathogens, whether or not typically skin-associated (i.e., PPS and PPNS microorganisms), occurred in only 1.0 and 18.9% of samples, respectively. Antibiotic-resistant bacteria, detected in 2.4% (*n* = 5) samples, included sulfamethoxazole-resistant *Pseudomonas putida* (*n* = 1), cephalosporin-resistant *Pseudomonas putida* (*n* = 2) and *Stenotrophomonas maltophilia* (*n* = 1), and methicillin-resistant *S. aureus* (*n* = 1).

### Host Factors Associated With Presence of Microorganisms

Ten of the 15 individual microorganism types had sufficient prevalence for statistical analysis, of which, three were associated with demographic/behavioral factors. Non-beta-hemolytic streptococci were less likely among participants who took public transit within the past week (*n* = 167) (6.6 vs. 20.5%, *p* = 0.01) or with team sport participation or a gym workout in the past week (*n* = 84) (4.1 vs. 16.9%, *p* < 0.01). Putative *Bacillus* spp. carriage was more likely among participants who had a pet (*n* = 150) (75.6 vs. 53.8%, *p* = 0.04) or swam in a pool in the past week (*n* = 45) (97.8 vs. 84.5%, *p* = 0.03). Mold carriage was more common among children (*n* = 103) than adults (*n* = 103) (66.0 vs. 34.9%, *p* < 0.01).

Regarding the four broader microbial categories, NPS and PPS microorganisms were unassociated with demographic/behavioral factors. NPNS microorganisms were more common among children than adults (98.1 vs. 88.3%, *p* = 0.01) and among participants who swam in a pool within the past week (100.0 vs. 91.3%, *p* = 0.04). PPNS microorganism were more common among males (*n* = 72) than females (*n* = 134) (27.8 vs. 14.2%, *p* = 0.02) and were less likely among children than adults (9.7 vs. 28.2%, *p* < 0.01). Additionally, antibiotic-resistant microorganisms (irrespective of category membership) were more common among adults with a high school degree or some college (*n* = 20) than those with at least an associate or technical degree (*n* = 83) (15.0 vs. 0.0%, *p* < 0.01).

### Generalized Linear Mixed-Effects Models

We used univariable and multivariable generalized linear mixed-effects models to assess associations between demographic/behavioral factors and microorganism categories with sufficient prevalence (NPS, NPNS, PPNS and antibiotic-resistant organisms) [S3]. In univariable analyses, NPNS microorganisms were significantly more likely among children (OR = 6.66, 95% CI: 1.45–30.55, *p* = 0.01), less likely among outdoor activity participants (OR = 0.10, 95% CI: 0.02–0.46, *p* < 0.01) and among those who used a topical skin product the day of the fair (OR = 0.03–0.91, *p* < 0.01). When combined in a multivariable model, adult/child status and participation in an outdoor activity remained significant predictors of NPNS microorganism carriage (Table 3).

**Table 3:** Questionnaire variables statistically significantly associated with microorganism categories in univariable/multivariable generalized linear mixed models for 206 Minnesota State Fair attendees (2014).

**Microorganism category**	**Associated questionnaire variable**	**Odds ratio (95% CI)**
Presumed non-pathogenic skin (NPS) microorganisms	Recent outdoor recreation (Yes vs. No)	0.07 (0.01, 0.88)^*^
Presumed non-pathogenic, non-skin (NPNS) microorganisms	Child vs.adult Adult Recent outdoor recreation (Yes vs. No)	6.61 (1.67–26.15)^*^ 0.10 (0.02–0.53)^**^
Potentially pathogenic skin (PPS) microorganisms	None	
Potentially pathogenic, non-skin (PPNS) microorganisms	Child vs. Adult	3.64 (1.67–7.96)^**^
Antibiotic-resistant organisms	None	

* *p < 0.05*,

** *p < 0.01*.

PPNS microorganisms were more likely among children (OR = 3.64, 95% CI: 1.67–7.96, *p* < 0.01) and males (OR = 2.33, 95% CI: 1.15–4.73, *p* = 0.02) (Table 3). When these variables were combined in a multivariable model, sex was no longer predictive of PPNS microorganisms, and the coefficient for adult/child changed by 9.5%, suggesting slight confounding by sex of the association of adult/child with PPNS microorganisms. An exploratory *X*^2^ analysis showed that females accounted for a significantly higher proportion of adults (72.8%) than children (57.3%) (*p* = 0.03); however, in the multivariable model an interaction term between adult/child status and sex was not statistically significant.

Given the importance of adult/child status as a predictor of NPNS and PPNS microorganisms, we examined the relationship between adult/child status and other demographic/behavioral factors using *X*^2^ tests. Notably, handwashing frequency was greater for adults than children (*p* < 0.01). However, handwashing frequency was not a significant univariable or multivariable predictor of carriage of either NPNS or PPNS microorganisms.

In the univariable assessment for NPS microorganisms, those who participated in an outdoor activity in the past week had significantly lower odds of carriage than those who had not (OR: 0.07, 95% CI: 0.01–0.88, *p* = 0.04), which was inconsistent with the results of the tabular analyses. Antibiotic-resistant microorganisms were associated with work with livestock (OR: 13.22, 95% CI 0.19–19.56, *p* = 0.05), but this analysis used only the subset of participants in the study who were employed.

## Discussion

This cross-sectional study sought to describe the types of microorganisms found on the hands of community-dwellers attending the Minnesota State Fair and to determine whether such carriage corresponds with demographic/behavioral factors. Among the 206 participants, the most common microorganisms were non-pathogenic, whether skin microbiota (NPS; 98.1% of participants) or not (NPNS; 93.2% of participants). The most prevalent NPNS microorganisms were *Bacillus* spp. (87.4% of participants), which occur in soil, a common exposure in the peri-domestic environment and on the State Fair grounds. Other common NPNS microorganisms were molds, which can be found in humid, dark, and damp areas, including soil with decomposing vegetation. Participants may have been exposed to molds at home through gardening or yard work or at any of several fairground sites, including animal barns (hay and straw) and landscaping, farming, and gardening exhibits.

Only two participants (1.0%) had detectable hand carriage of *S. aureus*, which in only one instance was MRSA (0.5%, 95% CI: 0.1–2.7%). The 2003–2004 National Health and Nutrition Examination Survey (NHANES) reported a MRSA prevalence of 1.5% (95% CI: 1.2–1.8%) from nasal swabs, and the National Institute for Occupational Safety and Health estimates a 1% general-population MRSA prevalence (40, 41). Our result aligns with these national estimates, despite some differences in sampling technique and population. Notably, we did not identify any pathogenic *E. coli*, which has been implicated in outbreaks at recent state and county fairs (42–44).

Although children were consistently more likely than adults to have detectable hand-source microorganisms, including various PPNS microorganisms, and although adults washed their hands more frequently than children, handwashing was unassociated with microorganism carriage. Unmeasured exposure differences between adults and children, as well as the variation in skin microbiota by age (45), may explain the observed differences in colonization by adult/child status.

Despite the differences in microorganisms carried by children and adults, most detected microorganisms were non-pathogenic, without associated concerns regarding community transmission either by behaviors participants engaged in at the State Fair, or activities in their daily lives. The Minnesota State Fair strongly encourages hand hygiene, in partnership with the Minnesota Department of Health. Handwashing stations are readily available throughout the fairgrounds, and promotional items, including signs and handheld fans that say, “I'm a fan of handwashing,” remind attendees to wash their hands. Conceivably, study participants' hand hygiene practices offered sufficient protection against carriage of pathogenic microorganisms. However, participants were less likely to assign themselves a “good” grade on handwashing at the State Fair than their usual practice. Perhaps their perception of poor hand hygiene practices at the State Fair actually made them more cautious of what they touched and when and how they washed their hands. As precedent for this phenomenon, nursing students who perceived higher risk of infection were more likely to exhibit appropriate hand hygiene practices than those who perceived a lower risk (46).

### Strengths and Limitations

This cross-sectional study used the Minnesota State Fair as a setting for assessing hand carriage of microorganisms by healthy individuals. Participants were willing to incorporate study activities, including biological sample collection, into their time at the State Fair. This model conceivably could be applied to other large community gatherings for recruiting into a range of point-prevalence or longitudinal studies. Indeed, since 2014 more than 103,000 Minnesota State Fair attendees have participated in research during their fair visit, evidence of this enrollment strategy's usefulness for many investigators (47).

Nonetheless, despite the potential for recruiting a wide range of State Fair attendees, participants in this study may not have been representative of the average fairgoer. The affiliation of the enrollment site with a university likely contributed to a sample that was more educated and health-conscious than the Minnesota population as a whole. Notably, 97.0% of study participants were from the seven-county Twin Cities metro area, overrepresenting the predominantly urban metro area relative to the total Minnesota population (48). Likewise, compared with U.S. Census estimates, participants also were more likely to be white (92.7 vs. 86.3%), Non-Hispanic (97.1 vs. 94.8%), and to have at least a bachelor's degree (73.8 vs. 34.8%). Moreover, 75.7% earned at least $50,000 per year, and 58.2% at least $75,000, whereas the statewide median income is $65,699 (49).

Because this was an exploratory study, and of the small sample size, we did not adjust the analyses for multiple comparisons. Future investigations of prevalence, characteristics, and epidemiology of microbial hand contamination in the community should consider using a larger sample size or narrower range of exposure variables to describe associations more fully. Additionally, we studied only hand carriage. Therefore, we cannot exclude colonization at other sites, which also may contribute to community transmission of microorganisms (50).

## Conclusions

Our study demonstrated the feasibility using a large community event to conduct a prevalence survey for microorganism carriage. The main findings were that (i) most participants carried non-pathogenic microorganisms, few carried pathogens, especially antimicrobial-resistant pathogens; (ii) adults and children differed with respect to both microorganism carriage and handwashing frequency; (iii) handwashing habits, behaviors at the fair and in daily life, were unassociated with microorganism carriage among community members.

## Data Availability Statement

The raw data supporting the conclusions of this article will be made available by the authors, without undue reservation.

## Ethics Statement

This study involving human participants was reviewed and approved by the University of Minnesota Institutional Review Board (No. 1406M1101). Written informed consent to participate in this study was provided by the participants or the participants' legal guardian/next of kin.

## Author Contributions

MM and BM conceived of the study idea. JJ and CC supported MM and BM in designing the protocols and survey materials. SJ, FN, MM, and BM collected the data. CC performed all laboratory procedures and reported prevalence results to the rest of the team. SJ, FN, MM, BM, and RB conducted the statistical analyses. MM and BM wrote the manuscript with significant editorial contributions from JJ. All authors contributed to the article and approved the submitted version.

## Conflict of Interest

The authors declare that the research was conducted in the absence of any commercial or financial relationships that could be construed as a potential conflict of interest.
