# Supplementary material for: Prevalence, Characteristics, and Epidemiology of Microbial Hand Contamination Among Minnesota State Fair Attendees (2014)
Source: Front Public Health. 2020 Dec 16;8:574444. doi: 10.3389/fpubh.2020.574444 (PMC7772179; doi:10.3389/fpubh.2020.574444)
Supplement: Supplementary file 1 [file Table_1.DOCX]

**Supplemental Table 1: Univariable Generalized Linear Mixed-effects Analyses of Questionnaire Data and Microorganism Categories from 206 Minnesota State Fair attendees (2014).**

|  | **NPS**  **Odds Ratio**  **(95% CI)** | **NPNS**  **Odds Ratio**  **(95% CI)** | **PPNS**  **Odds Ratio**  **(95% CI)** | **Antibiotic Resistant Odds Ratio**  **(95% CI)** |
| --- | --- | --- | --- | --- |
| **Child < 18 years old v. Adult ≥ 18 years old** | 0.50 (0.04, 5.55) | 6.66 (1.45, 30.55) * | 3.64 (1.67, 7.96) ** | 0.66 (0.11, 4.04) |
| **Female v. Male** | 0.93 (0.08, 10.43) | 0.29 (0.06, 1.34) | 2.33 (1.15, 4.73) * | 0.35 (0.06, 2.14) |
| **Race**  Asian  Black/African American  White  More than one race  Unknown/not reported | -- | -- | -- | -- |
| **Ethnicity Hispanic v. Non-Hispanic** | -- | -- | -- | -- |
| **Residence Metro Area v. Not Metro Area (n=202)** | -- | -- | -- | -- |
| **Sites visited /Activities at the fair**  *Animal building v. no animal building*  *Restrooms v. no restrooms at fair*  *Food vendor v. no food vendor*  *Touched animals v. didn’t touch animals* | --  0.63 (0.06, 7.03)  --  -- | 1.88 (0.51, 6.96)  0.32 (0.09, 1.19)  0.71 (0.19, 2.66)  1.02 (0.27, 3.85) | 1.02 (0.49, 2.13)  1.17 (0.58, 2.38)  1.56 (0.67, 3.64)  1.30 (0.59, 2.92) | --  0.52 (0.08, 3.18)  1.51 (0.16, 13.77)  0.89 (0.10, 8.19) |
| **Own a pet** | -- | 1.53 (0.48, 4.86) | 0.94 (0.43, 2.04) | 0.55 (0.10, 3.39) |
| **Time since last handwashing ≥ 2 hours v. < 2 hours**  **(n=203)** | 0.27 (0.02, 3.07) | 2.13 (0.58, 7.93) | 1.01 (0.49, 2.08) | 1.20 (0.20, 7.36) |
| **Most recent handwashing was at the fair v. not at the fair** | 2.61 (0.23, 29.29) | 0.70 (0.23, 2.16) | 1.01 (0.50, 2.03) | 0.51 (0.08, 3.11) |
| **Most recent handwashing method Alcohol-based sanitizer v. Soap and water (n=203)** | 0.11 (0.01, 1.25) | 0.43 (0.12, 1.53) | 0.88 (0.31, 2.09) | -- |
| **Handwashing frequency per day (n = 199)**  ≤ 5 times  6-10 times  > 10 times | --  -- | ref.  1.48 (0.34, 6.46)  0.48 (0.14, 1.68) | ref.  1.18 (0.52, 2.69)  1.04 (0.41, 2.64) | --  -- |
| **Applied a topical skin product** | -- | 0.16 (0.03, 0.91) * | -- | -- |
| **In the past week they…**  *Participated in individual sports*  *Worked out at a gym or did team sports*  *Attended another social or community event*  *Swam in a pool*  *Swam in a lake*  *Gardened*  *Used public transit*  *Outdoor recreation (hike/camp)* | 1.47 (0.13, 16.48)  --  1.00 (0.09, 11.31)  --  --  --  2.17 (0.19, 24.57)  0.07 (0.01, 0.88) * | 2.85 (0.77, 10.55)  1.23 (0.40, 3.81)  0.90 (0.29, 2.80)  --  --  --  0.69 (0.15, 3.25)  0.10 (0.02, 0.46) ** | 0.63 (0.30, 1.31)  1.34 (0.67, 2.71)  0.74 (0.34, 1.59)  1.09 (0.48, 2.51)  1.15 (0.40, 3.28)  --  0.61 (0.27, 1.39)  -- | 0.91 (0.15, 5.56)  0.99 (0.16, 6.04)  3.07 (0.50, 18.81)  --  --  --  0.93 (0.10, 8.58)  -- |
| **Self or child in daycare** | 0.29 (0.03, 3.36) | 0.52 (0.13, 2.05) | 1.61 (0.63, 4.12) | -- |
| **During occupation/regular volunteer activity, they…**  *Work with livestock*  *Work in clinic*  *Work in daycare or childcare facility*  *Wear gloves regularly* | --  --  --  -- | --  0.43 (0.08, 2.26)  1.02 (0.26, 4.04)  0.88 (0.18, 4.35) | 6.63 (0.87, 50.40)  --  1.15 (0.38, 3.53)  0.29 (0.04, 2.35) | 13.22 (1.05, 167.09) *  --  --  1.93 (0.19, 19.56) |

* p < 0.05, ** p < 0.01

*Italics indicate non-mutually exclusive responses*

*--* indicates insufficient variability in data to use generalized linear mixed models
