# Supplementary material for: Prevalence, Characteristics, and Epidemiology of Microbial Hand Contamination Among Minnesota State Fair Attendees (2014)
Source: Front Public Health. 2020 Dec 16;8:574444. doi: 10.3389/fpubh.2020.574444 (PMC7772179; doi:10.3389/fpubh.2020.574444)
Supplement: Supplementary file 2 [file Presentation_1.pdf]

<u>Minnesota Munchkins with Microbes</u>
<u>Step-by-Step Study Process</u>

1. Welcome & Eligibility

a. Tell them about the purpose of the study: *We are examining the different types of bacteria on the hands of children and their family members to look at similarities of the bacteria types within families, and then across different families in the community. The project would involve about 15-20 minutes of your time, and consists of a hand swab, pressing your hand into a petri dish, and a survey about your daily activities as well as your time here at the State Fair. [[This project is designed to help us understand what environmental factors may be associated with the presence of certain types of bacteria, both harmless types and those that can potentially cause disease.]]*

b. Fill out an eligibility form with the family if they are interested in participating in the study. <u>The form has their family ID # at the top</u>

c. <u>**GOPHER KIDS STUDY**</u>: Be sure to ask Question #5 on the eligibility form. If a family is a Gopher Kids Study family, and have visited the GKS group already, they should have an index card with their <u>GKS ID</u> listed on it. Take that card, and write the M^3 ID on the card as well. <u>GIVE THIS CARD TO BOZENA OR MEGHAN</u>. If the family says that they are a GKS participating family but have not yet been to the GKS group, <u>get an index card, write their M^3 ID on it, and tell them to give the card to the GKS staff when they go to that station.</u>

2. Obtaining Consent/Assent

a. Review the consent/assent form with participants, give them a copy. [[We are not having them sign the forms so that records remain anonymous.]] Ask each adult the following questions

and re-explain the study as needed until they understand the answers listed below:

i. What will happen if you decide to not participate in this study? *Appropriate responses:*
   1. Nothing
   2. We will not be eligible for the study
   3. We will not have anything done at this booth

ii. Can you explain back to me what you expect to do as part of your involvement in this study? *Appropriate responses:*
   1. Hand swabs
   2. Hand in agar plate
   3. Survey

iii. What is the compensation for this study? *Appropriate responses:*
   1. Participation in a drawing
   2. Target card & Giant Microbe
      a. If participants provide the second response, they will be asked whether every participant receives the compensation to which they will need to understand the answer is no, only 6 families over the two days will receive the compensation.

3. Move to the Microbiology “Station” for sample collection

PUT ON A NEW PAIR OF GLOVES FOR EACH <u>PARTICIPANT</u>

4. Plate Participant’s Dominant Hand
   a. Ask what hand is the dominant hand. (Writing hand for little kids – if they can’t write assume right hand.)
   b. Write the participant ID on the label and stick it on the petri dish
   c. Open the plate and stick the participant’s thumb in the dish in a “thumbs up” position and apply pressure from fingernail to

palm. Follow the curvature of the plate as best as possible to save space.

d. Take the four fingers side-by-side and apply pressure at fingertips. Have participant side top part of fingers over the edge and then apply pressure at the knuckles to get top of palm surface.
e. Twist hand slightly inward to apply pressure on the fleshy part of the outside palm area.

*****Please ensure that you don't break the surface of the media, i.e. that participants don't press their nails into the gel. (This creates indents where colonies can grow, but are difficult to distinguish and therefore, not helpful to the people doing the culture analysis.*****

f. SPREAD THE SAMPLE ACROSS THE PLATE
    i. Light spreading motion in 1 direction (top to bottom) on the length of the plate.
    ii. Turn the plate 90 degrees and repeat the same light spreading motion from top to bottom.

g. Dispose of spreader
h. Place petri dish in cooler

5. Swab non-dominant hand
    a. RECORD TUBE NUMBER ON ELIGIBILITY FORM
    b. Un-wrap swab
    c. Dip in saline buffer (large tubes)
    d. Swab the participant's non-dominant (non-writing) hand for 30 second over the entire palm, fingers, and between fingers beginning on the outside of the thumb, surface of thumb, in a zig-zag motion across the palm and finger surfaces collecting from webbed part of fingers along the way.
    e. Open 2mL tube
    f. Break swab into tube
    g. Close tube

h. PUT TUBE IN FREEZER ASAP
i. Dispose of trash.

6. ****DISPOSE OF GLOVES****

7. **Move to Survey "Station" and Complete Survey**
    a. Select adult/child survey
    b. Ask corresponding questions
    c. If possible, do child's and parent's side-by-side with other staff member

8. **Wrap up!**

9. The family will receive a drawing form to fill out to win Target Card & Giant Microbes.

10. While the family is filling out the form, take a moment to make sure you have done all of the following:
    a. Eligibility form has a study ID on it
    b. Family has been asked if they are part of Gopher Kids Study and given a card with M^3 ID # as needed
    c. Eligibility form ID matches survey ID
    d. Petri dish sample has been taken and <u>labeled</u>
    e. Swab has been taken and <u>tube number was recorded</u>
    f. Family has copy of consent/assent forms

11. **THANK THE FAMILY!!!**

Get ready for the next family!!! ☺

If you are unsure of anything or questions come up in the process, just ask Bozena & Meghan.
