# Supplementary material for: Prevalence, Characteristics, and Epidemiology of Microbial Hand Contamination Among Minnesota State Fair Attendees (2014)
Source: Front Public Health. 2020 Dec 16;8:574444. doi: 10.3389/fpubh.2020.574444 (PMC7772179; doi:10.3389/fpubh.2020.574444)
Supplement: Supplementary file 3 [file Presentation_2.pdf]

Minnesota Munchkins With Microbes Survey

Please complete the survey below.

Thank you!

This person is an adult:
◯ True
◯ False
(If child make sure it skips to child questions)

Minnesota Munchkins with Microbes ID Household ID_Adult/Child_Male/Female_Oldest First
____
(000_A/C_M/F_00)

Family Code
____
(000-999)

Demographic Information

Gopher Kids Study Participant?
◯ Yes
◯ No

Gopher Kids Study Participant?
◯ Yes
◯ No

ID Comments
(List other family members participating (i.e. with Mom, w

ID Comments
(List other family members participating (i.e. with Mom, w

Date subject signed consent
____
(MM-DD-YYYY)

Date subject gave assent
____
(MM-DD-YYYY)

Race
◯ American Indian/Alaska Native
◯ Asian
◯ Native Hawaiian or Other Pacific Islander
◯ Black or African American
◯ White
◯ More Than One Race
◯ Unknown / Not Reported

Race
◯ American Indian/Alaska Native
◯ Asian
◯ Native Hawaiian or Other Pacific Islander
◯ Black or African American
◯ White
◯ More Than One Race
◯ Unknown / Not Reported

Ethnicity

◯ Hispanic or Latino ◯ NOT Hispanic or Latino ◯ Unknown / Not Reported

Ethnicity

◯ Hispanic or Latino ◯ NOT Hispanic or Latino ◯ Unknown / Not Reported

Gender
◯ Female
◯ Male
◯ Trans
◯ Prefer not to disclose

Gender
◯ Female
◯ Male
◯ Trans
◯ Prefer not to disclose

Date of birth _______________________________

Date of birth _______________________________

Age (years) _______________________________

Age (years) _______________________________
(If >= 18 years old use adult survey)

Zip Code _______________________________
(5 digit zip)

How close is your nearest neighbor?

- ☐ 0 ft - live in an apartment/condo/multiplex
- ☐ 5-100 ft - urban single home
- ☐ 100 ft - 1/2 mi - suburban
- ☐ 1/2 mi - 2 mi - rural
- ☐ 2mi + very rural

What type of potable water do you use? (out of faucet)

- ○ City water
- ○ Well water
- ○ Other water source

If other above, describe: __________________________________

Education

- ○ Did not finish high school
- ○ High school graduate
- ○ Some college
- ○ College graduate (Technical/Associate)
- ○ College graduate (Bachelor)
- ○ Graduate or Professional School

What grade will you enter this fall? (Or are presently in if school has already started)

- ○ K
- ○ 1st
- ○ 2nd
- ○ 3rd
- ○ 4th
- ○ 5th
- ○ 6th
- ○ 7th
- ○ 8th
- ○ 9th
- ○ 10th
- ○ 11th
- ○ 12th

Income

- ○ Less than $10,000
- ○ $10,000 to $14,999
- ○ $15,000 to $24,999
- ○ $25,000 to $34,999
- ○ $35,000 to $49,999
- ○ $50,000 to $74,999
- ○ $75,000 to $99,999
- ○ $100,000 to $149,999
- ○ $150,000 to $199,999
- ○ $200,000 or more

Fair Questions

Select the time closest to when you arrived at the Fair today.

- ◯ 06:00 AM
- ◯ 07:00 AM
- ◯ 08:00 AM
- ◯ 09:00 AM
- ◯ 10:00 AM
- ◯ 11:00 AM
- ◯ 12:00 PM
- ◯ 01:00 PM
- ◯ 02:00 PM
- ◯ 03:00 PM
- ◯ 04:00 PM
- ◯ 05:00 PM
- ◯ 06:00 PM
- ◯ 07:00 PM
- ◯ 08:00 PM

Select the time closest to when you arrived at the Fair today.

- ◯ 06:00 AM
- ◯ 07:00 AM
- ◯ 08:00 AM
- ◯ 09:00 AM
- ◯ 10:00 AM
- ◯ 11:00 AM
- ◯ 12:00 PM
- ◯ 01:00 PM
- ◯ 02:00 PM
- ◯ 03:00 PM
- ◯ 04:00 PM
- ◯ 05:00 PM
- ◯ 06:00 PM
- ◯ 07:00 PM
- ◯ 08:00 PM

What places at the Fair have you already visited? (Check all that apply)

- ☐ Non-animal building (i.e. Horticulture, Education building, Eco-experience)
- ☐ Horse Barn
- ☐ Swine Barn
- ☐ Cattle Barn
- ☐ Sheep & Poultry Barn
- ☐ Miracle of Birth Barn
- ☐ Grandstand
- ☐ Kidway rides
- ☐ Midway rides
- ☐ Food Vendor
- ☐ Other Vendor Stands (i.e. media tents, Heritage Square, International Bazaar)
- ☐ ATM
- ☐ Medical Station
- ☐ Restroom
- ☐ D2D building
- ☐ Other

What places at the Fair have you already visited? (Check all that apply)

- ☐ Non-animal building (i.e. Horticulture, Education building, Eco-experience)
- ☐ Horse Barn
- ☐ Swine Barn
- ☐ Cattle Barn
- ☐ Sheep & Poultry Barn
- ☐ Miracle of Birth Barn
- ☐ Grandstand
- ☐ Kidway rides
- ☐ Midway rides
- ☐ Food Vendor
- ☐ Other Vendor Stands (i.e. media tents, Heritage Square, International Bazaar)
- ☐ ATM
- ☐ Medical Station
- ☐ Restroom
- ☐ D2D Building
- ☐ Other

If "Other" sites visited ______________________________

If "Other" sites visited ______________________________

What was your most recent stop before visiting the D2D building?

- ◯ Non-animal building (i.e. Horticulture, Education building, Eco-experience)
- ◯ Horse Barn
- ◯ Swine Barn
- ◯ Cattle Barn
- ◯ Sheep & Poultry Barn
- ◯ Miracle of Birth Barn
- ◯ Grandstand
- ◯ Kidway rides
- ◯ Midway rides
- ◯ Food Vendor
- ◯ Other Vendor Stands (i.e. media tents, Heritage Square, International Bazaar)
- ◯ ATM
- ◯ Medical Station
- ◯ Restroom
- ◯ Other

What was your most recent stop before visiting the D2D building?

- ◯ Non-animal building (i.e. Horticulture, Education building, Eco-experience)
- ◯ Horse Barn
- ◯ Swine Barn
- ◯ Cattle Barn
- ◯ Sheep & Poultry Barn
- ◯ Miracle of Birth Barn
- ◯ Grandstand
- ◯ Kidway rides
- ◯ Midway rides
- ◯ Food Vendor
- ◯ Other Vendor Stands (i.e. media tents, Heritage Square, International Bazaar)
- ◯ ATM
- ◯ Medical Station
- ◯ Restroom
- ◯ Other

If "Other" site was most recently visited ______________________________

If "Other" site was most recently visited ______________________________

Have you touched any animals at the fair yet today?

- ◯ Yes
- ◯ No

Have you touched any animals at the fair yet today?

- ◯ Yes
- ◯ No

If "yes", touched animals, which animals?

- ☐ Cow
- ☐ Sheep
- ☐ Horse
- ☐ Pig
- ☐ Chicken/Duck
- ☐ Alpacas (Llamas)
- ☐ Goats
- ☐ Dogs
- ☐ Rabbits
- ☐ Other

If "yes", touched animals, which animals?

- ☐ Cow
- ☐ Sheep
- ☐ Horse
- ☐ Pig
- ☐ Chicken/Duck
- ☐ Alpacas (Llamas)
- ☐ Goats
- ☐ Dogs
- ☐ Rabbits
- ☐ Other

If "other" animal type: ______________________________

If "other" animal type: ______________________________

Antibiotic Use

Been treated with antibiotics

◯ Yes, for the past 6 months (long-term daily use)
◯ Yes, within the past 6 months
◯ Yes, within the past 1 month
◯ No, not within the past 6 months
(This may include use as anti-malarial medication, acne, prophylaxis from dog bite, etc.)

Did you receive any pills or syrup from the doctor within the past 6 months?

◯ Yes
◯ No
◯ Unsure
(This may include use as anti-malarial medication, acne, prophylaxis from dog bite, etc.)

If "unsure" for the dates/duration of usage write what information you have. i.e. "during school", "during winter", etc.

____

If treated with antibiotics within past 6 months, what was the reason? i.e. strep throat, pink eye, UTI, wound infection

____
(This may include use as anti-malarial medication, acne, prophylaxis from dog bite, etc.)

If yes, determine what the medicine was used for... Sore throat Fever Coughing Pink Eye etc.

____
(This may include use as anti-malarial medication, acne, prophylaxis from dog bite, etc.)

If yes, do you know whether the medicine was an antibiotic?

◯ Yes, antibiotic
◯ No, not antibiotic
◯ Unsure

What have you applied to your skin so far today?

☐ Makeup
☐ Sunscreen
☐ Insect repellent
☐ Topical acne products
☐ Other

If "yes" to topical acne product, what type of product? Gel, cream, cleanser, name brand if they can remember

____

If "other" above, list information here:

____

What have you applied to your skin so far today?

☐ Makeup
☐ Sunscreen
☐ Insect repellent
☐ Topical acne products
☐ Other

If "yes" to topical acne product, what type of product? Gel, cream, cleanser, name brand if they can remember

____

If "other" above, list information here:

____

Hand Hygiene

When was the last time you washed or cleaned your hands?

- ◯ < 30 minutes ago
- ◯ 30 minutes - 2 hours ago
- ◯ 2 hours - 4 hours ago
- ◯ 4 hours - 6 hours ago
- ◯ 6 hours - 8 hours ago
- ◯ more than 8 hours ago

When was the last time you washed or cleaned your hands?

- ◯ < 30 minutes ago
- ◯ 30 minutes - 2 hours ago
- ◯ 2 hours - 4 hours ago
- ◯ 4 hours - 6 hours ago
- ◯ 6 hours - 8 hours ago
- ◯ more than 8 hours ago

Where did you last wash or clean your hands?

- ◯ Public restroom at the Fair
- ◯ Hand wash station at the Fair
- ◯ Public restroom NOT at the Fair
- ◯ At home
- ◯ At work
- ◯ In the car
- ◯ Other

Where did you last wash or clean your hands?

- ◯ Public restroom at the Fair
- ◯ Hand wash station at the Fair
- ◯ Public restroom NOT at the Fair
- ◯ At home
- ◯ At work
- ◯ In the car
- ◯ Other

If "other" above... ______________________________

If "other" above... ______________________________

What did you last use to clean your hands?

- ◯ Water
- ◯ Soap and water
- ◯ Alcohol-based sanitizer
- ◯ Wet wipe
- ◯ Napkin
- ◯ Pants/shorts
- ◯ Other

What did you last use to clean your hands?

- ◯ Water
- ◯ Soap and water
- ◯ Alcohol-based sanitizer
- ◯ Wet wipe
- ◯ Napkin
- ◯ Pants/shorts
- ◯ Other

If "other" above... ______________________________

If "other" above... ______________________________

When was the last time you used the bathroom?

- ◯ < 30 minutes ago
- ◯ 30 minutes - 2 hours ago
- ◯ 2 hours - 4 hours ago
- ◯ 4 hours - 6 hours ago
- ◯ 6 hours - 8 hours ago
- ◯ More than 8 hours ago

When was the last time you used the bathroom?
- ◯ < 30 minutes ago
- ◯ 30 minutes - 2 hours ago
- ◯ 2 hours - 4 hours ago
- ◯ 4 hours - 6 hours ago
- ◯ 6 hours - 8 hours ago
- ◯ More than 8 hours ago

Where did you last use the bathroom
- ◯ At Home
- ◯ State Fair
- ◯ Friend or Relative's House
- ◯ Other

Where did you last use the bathroom?
- ◯ At Home
- ◯ State Fair
- ◯ Friend or Relative's House
- ◯ Other

Did you wash or clean your hands after using the bathroom?
- ◯ Yes
- ◯ No

Did you wash or clean your hands after using the bathroom?
- ◯ Yes
- ◯ No

If yes, with what did you clean your hands after using the bathroom?
- ◯ Water
- ◯ Soap and water
- ◯ Alcohol-based sanitizer
- ◯ Wet wipe
- ◯ Napkin
- ◯ Pants/shorts/shirt
- ◯ Other

If yes, with what did you clean your hands after using the bathroom?
- ◯ Water
- ◯ Soap and water
- ◯ Alcohol-based sanitizer
- ◯ Wet wipe
- ◯ Napkin
- ◯ Pants/shorts/shirt
- ◯ Other

Have you had to change someone's diaper today? i.e. a child, sibling, friend's child, etc.
- ◯ < 30 minutes ago
- ◯ 30 minutes - 1 hour ago
- ◯ 1 hour - 2 hours ago
- ◯ More than 2 hours ago
- ◯ N/A I did not have to change a child's diaper

If the participant has changed a diaper... Did you wash or clean your hands after changing the diaper?
- ◯ Yes
- ◯ No

If yes, with what did you clean your hands after changing the diaper?
- ◯ Water
- ◯ Soap and water
- ◯ Alcohol-based sanitizer
- ◯ Wet wipe
- ◯ Napkin
- ◯ Pants/shorts/shirt
- ◯ Other

Around the house, what do you usually use to wash your hands?
- ◯ Water
- ◯ Soap and water
- ◯ Alcohol-based sanitizer
- ◯ Wet wipe
- ◯ Other

If "other" for washing hands around house... ______________________________

If "other" for washing hands around house... ______________________________

Do you own any of the following animals?

- ☐ Dog
- ☐ Cat
- ☐ Bird
- ☐ Fish
- ☐ Hamster/Gerbil
- ☐ Cow
- ☐ Horse
- ☐ Sheep
- ☐ Goat
- ☐ Pig
- ☐ Rabbit
- ☐ Poultry
- ☐ Reptiles/Amphibians
- ☐ Other

Do you own any of the following animals?

- ☐ Dog
- ☐ Cat
- ☐ Bird
- ☐ Fish
- ☐ Hamster/Gerbil
- ☐ Cow
- ☐ Horse
- ☐ Sheep
- ☐ Goat
- ☐ Pig
- ☐ Rabbit
- ☐ Poultry
- ☐ Reptiles/Amphibians
- ☐ Other

If own "other" animal ______________________________

If own "other" animal ______________________________

What type of farming do you participate in?

- ☐ None
- ☐ Soybeans
- ☐ Corn
- ☐ Other crops
- ☐ Pigs
- ☐ Cows
- ☐ Poultry
- ☐ Other animals

What type of farming does your family participate in?

- ☐ None
- ☐ Soybeans
- ☐ Corn
- ☐ Other crops
- ☐ Pigs
- ☐ Cows
- ☐ Poultry
- ☐ Other animals

Workplace Exposures

We would like to ask you about the conditions of your current and recent (within the past 30 days) employment and volunteer activities. Please indicate which of the following best characterize your working conditions (mark all that apply):

- ☐ Frequent (3+ times per week) physical contact with livestock
- ☐ Frequent (3+ times per week) physical contact with livestock feed/housing/supplies
- ☐ Frequent (3+ times per week) physical contact with domestic animals, e.g. dogs, cats
- ☐ Frequent (3+ times per week) physical contact with patients in an inpatient clinical setting
- ☐ Work in an inpatient clinical setting
- ☐ Frequent (3+ times per week) physical contact with patients in an outpatient clinical setting
- ☐ Work in an outpatient clinical setting
- ☐ Work in a daycare / childcare facility
- ☐ Works in an educational setting (high school or lower)
- ☐ None of these apply to me

Do you have a job or volunteer at a specific location at least once per week?

- ◯ Yes
- ◯ No

(If "No" skip this page)

We would like to ask you about the conditions of your current and recent (within the past 30 days) employment and volunteer activities. Please indicate which of the following best characterize your working conditions (mark all that apply):

- ☐ Frequent (3+ times per week) physical contact with livestock
- ☐ Frequent (3+ times per week) physical contact with livestock feed/housing/supplies
- ☐ Frequent (3+ times per week) physical contact with domestic animals, e.g. dogs, cats
- ☐ Frequent (3+ times per week) physical contact with patients in an inpatient clinical setting
- ☐ Work in an inpatient clinical setting
- ☐ Frequent (3+ times per week) physical contact with patients in an outpatient clinical setting
- ☐ Work in an outpatient clinical setting
- ☐ Work in a daycare / childcare facility
- ☐ Works in an educational setting (high school or lower)
- ☐ None of these apply to me.

Do you wear gloves regularly at your job or volunteer location?

- ◯ Yes
- ◯ No

Do you wear gloves regularly at your job or volunteer location?

- ◯ Yes
- ◯ No

Best estimate for number of times washing your hands per day (including at work).

- ◯ 0
- ◯ 1-2
- ◯ 3-5
- ◯ 6-10
- ◯ 10-15
- ◯ 15+

Best estimate for number of times washing your hands per day (including at work).

- ◯ 0
- ◯ 1-2
- ◯ 3-5
- ◯ 6-10
- ◯ 10-15
- ◯ 15+

Recreation

At least once over the course of the past week, I have attended or participated in:

- ☐ Individual sports, e.g. running, biking
- ☐ Gym / fitness center / climbing facility
- ☐ Team sports, e.g. basketball, football
- ☐ Community events, e.g. indoor/outdoor concert, Twins game, art fair
- ☐ Other social events, i.e. dancing, social parties
- ☐ Swimming in a pool
- ☐ Swimming in a lake/river
- ☐ Taken public transportation

At least once over the course of the past week, I have attended or participated in:

- ☐ Individual sports, e.g. running, biking
- ☐ Gym / fitness center / climbing facility
- ☐ Team sports, e.g. basketball, football
- ☐ Community events, e.g. indoor/outdoor concert, Twins game, art fair
- ☐ Other social events, i.e. dancing, social parties
- ☐ Swimming in a pool
- ☐ Swimming in a lake/river
- ☐ Taken public transportation

Any other recreational activities of note: ______________________________

Any other recreational activities of note: ______________________________

Family Handwashing

Did/do you use any tools to help your children learn about hand washing? (Check all that apply)

- ☐ None
- ☐ Singing the alphabet song or happy birthday
- ☐ Using a reward for good hand washing
- ☐ Hanging pictures on how to properly hand wash
- ☐ Other
- ☐ N/A do not have children

If "other" above: ______________________________

Did you remember learning to wash your hands by using any of the following tricks? (Check all that apply)

- ☐ None
- ☐ Singing the alphabet song or happy birthday
- ☐ Using a reward for good hand washing
- ☐ Hanging pictures on how to properly hand wash
- ☐ Other

If "other" above: ______________________________

Do your children attend day care or receive care outside of the home?

- ◯ Yes
- ◯ No

Do you go to daycare or someone's home where you play with other kids during the day?

- ◯ Yes
- ◯ No

Size of child(ren)'s day care

- ◯ 0-5 children
- ◯ 5-15 children
- ◯ 15-40 children
- ◯ 40+ children

Notes re: daycare ______________________________

Notes re: daycare ______________________________

Do your children attend school including home schooling, non-traditional school, and/or other forms of formal education?

- ◯ Yes
- ◯ No

Do you go to school?

- ◯ Yes, outside of the home
- ◯ Yes, at home
- ◯ No, not yet!

Notes re: children in school ______________________________

Notes re: school ______________________________

Additional Questions & Notes

If you had to give yourself a grade for hand washing practices in general, you would say:
- ◯ A
- ◯ B
- ◯ C
- ◯ D
- ◯ F

If you had to give yourself a grade for hand washing practices today, you would say:
- ◯ A
- ◯ B
- ◯ C
- ◯ D
- ◯ F

If you had to give your child(ren) a grade for hand washing practices in general, you would say:
- ◯ A
- ◯ B
- ◯ C
- ◯ D
- ◯ F

If you had to give your child(ren) a grade for hand washing practices today, you would say:
- ◯ A
- ◯ B
- ◯ C
- ◯ D
- ◯ F

If you had to give yourself a grade for hand washing practices in general, you would say:
- ◯ A
- ◯ B
- ◯ C
- ◯ D
- ◯ F

If you had to give yourself a grade for hand washing practices today, you would say:
- ◯ A
- ◯ B
- ◯ C
- ◯ D
- ◯ F

Tell us two adjectives/descriptors about your day so far at the State Fair: ______________________________

Tell us two adjectives/descriptors about your day so far at the State Fair: ______________________________
